# Characteristics of the Frontier Extended Stay Clinic: a new facility model

**DOI:** 10.3402/ijch.v72i0.21344

**Published:** 2013-08-05

**Authors:** Rosyland Frazier, Sanna Doucette

**Affiliations:** Institute of Social and Economic Research, University of Alaska Anchorage, AK, USA

**Keywords:** access, emergency care, extended-stay encounter, Frontier Extended Stay Clinic, health services delivery, rural health

## Abstract

**Purpose:**

In 2004, 5 remote clinics – 4 in rural frontier communities in Alaska and 1 in Washington – were funded to pilot and examine the effectiveness and appropriateness of a new facility model. Transporting patients from these locations to higher levels of care is not always possible requiring these facilities to expand their scope of services and provide care for extended periods. The Frontier Extended Stay Clinic (FESC) model is staffed and equipped to provide the combined services usually found in the separate settings of an outpatient primary-care clinic, inpatient acute care hospital and emergency room. This is a descriptive study of the characteristics of these pilot facilities and an analysis of patient utilization and outcomes.

**Methods:**

The 5 clinics collected outcome data for 2,226 extended-stay encounters of 4 hours or longer from 15 September 2005 to 14 September 2010. Data from these extended-stay encounters were summarized, and descriptive statistics were used to describe: number and duration of encounters, when the encounters started, chief compliant, discharge diagnoses, transfer destination, Medicare and Medicaid eligibility, and type of encounter.

**Findings:**

From 2005 to 2010, the mean duration of an extended-stay encounter was 9.1 hours. All of the clinics experienced many extended-stay encounters that were initiated or continued after normal business hours. The 5 most frequent diagnoses at discharge for extended encounters were cardiovascular, gastrointestinal, injury, substance abuse and pneumonia/bronchitis. Almost half, 47.6%, of extended-stay encounters resulted in discharge of the patient without a need for either non-urgent follow-up referral or transport. Extended-stay encounters that ended in a patient being transported to another medical facility were 43.7% of the total. More than a quarter (26.9%) of extended-stay encounters were eligible for Medicare payment.

**Conclusion:**

While many of communities can support a facility for primary care, there is an on-going need for facilities in remote frontier areas to also provide emergent and extended-stay care. The FESC can provide access to primary, emergent and extended-stay services in these locations.

In the 1990s, health services delivery offered limited flexibility to meet the health care needs of rural regions and frontier communities. Frontier advocates – including health officials in the State of Alaska, several state Offices of Rural Health, Primary Care Offices and Primary Care Associations, and their congressional representatives – thought that a facility that is more than a primary-care clinic but less than a hospital would meet the need in some areas. Thus began the exploration of a new facility type or other mechanism that would enable reimbursement of expanded primary-care services (1). The concept of a Frontier Extended Stay Clinic (FESC) was proposed as a facility that would be eligible for reasonable cost-based reimbursement and provide a higher intensity and expanded scope of services than that of a traditional primary-care clinic. This new model would *build up capacity* within a clinic to a more advanced type of provider rather than to *downsize from a hospital*. The FESC concept combines service found in an outpatient primary-care clinic, the inpatient acute care hospital and the emergency room.

## Background

The initial public recognition of a clinic of this type was in introduction of Senate Bill 1342 – the Medicare Frontier Health Clinic and Center Act of 1997 by Senators Frank Murkowski of Alaska and Craig Thomas of Wyoming – more commonly known as the “frontier super clinic” bill. Congressional action continued in 1999 to move from the “frontier super clinic” to the Extended Stay Primary Care Clinic when Alaska Senator Ted Stevens, Chairman of the Senate Appropriations Committee, inserted language concerning Extended Stay Primary Care into its report. In 2000, as a result of the congressional committee's report, the Health Resources Services Administration (HRSA) Office of Rural Health Policy (ORHP) contracted with the National Center for Frontier Communities (formerly named Frontier Education Center) to determine the need and interest in an Extended Stay Primary Care Clinic program (2). In 2003, there were 2 Congressional actions to encourage the development of this new facility type – the FESC. The first was the Medicare Modernization Act of 2003, which gave authority to the Centers for Medicare and Medicaid Services (CMS) at the Department of Health and Human Services (DHHS) to conduct a demonstration to reimburse extended-stay care received by Medicare beneficiaries. The second action in the Consolidations Acts of 2004, 2005 and 2006 included the funding for the HRSA ORHP “to examine the effectiveness and appropriateness of a new type of provider, the FESC, in providing health-care services in certain remote locations” (3).

In 2005, the SouthEast Alaska Regional Health Consortium (SEARHC) was awarded funds from HRSA through a cooperative agreement to conduct a program to examine the effectiveness and appropriateness of a new “model” of care – FESC – in providing the combination of primary, emergency and inpatient health care services in certain remote areas in Alaska and Washington. The 5 clinic sites that participated in this demonstration were the Haines Medical Center (HMS) in Haines, Alaska and the Alice Roberts Medical Center (ARMC) in Klawock, Alaska (both of which are a part of SEARHC); lliuliuk Family and Health Services (IFHS) in Unalaska, Alaska; Cross Road Medical Center (CRMC) in Glennallen, Alaska; and Inter Island Medical Center (IIMC) in Friday Harbor, Washington.

Providers or facilities that combine clinic and hospital services in rural and frontier areas are not a new concept. Australia has been working towards a definition of remote health that reflects a similar need to define frontier in the context of this “New Provider Type.” The 2 approaches to defining remoteness include – geographical isolation (as defined by the 75 miles from the next level of care for a FESC facility) and practice approaches (4). Looking at the historical development of emergency centres, a trauma surgeon determined that “… the best decision for a given patient depends on the patient's specific injuries, the hospital resources, geography, weather, and length and mode of transport available.” … “The best system for a given community or region is one that begins with a triage scheme that is evidence-based to the greatest extent possible but is then modified based on community or regional resources and geography” (5). This supports the need to organize health care delivery relative to the geography and resources. Patients like to receive services close to home. The same is true with those who live in remote areas. In Australia, a study of Aboriginal people examines the stress and discomfort that is created when people must travel away from their home community to receive care. There is the additional transportation cost, accommodation issues, separation from family members and their support. Also, the potential impact on the quality of care brought about by misunderstandings due to language and cultural differences. These reasons help explain the unwillingness of remote and frontier residents that have to travel at a time of illness to seek higher levels of care that can perhaps be provided in their home community (6).

## Methods

Health Resources Services Administration (HRSA) Office of Rural Health Policy (ORHP) required the FESCs to be located at least 75 miles from a hospital, placing them in geographic isolation and serving small populations. The distance to higher levels of care and difficulties with patient transportation require these clinics to provide urgent and emergent care. Patients may need to be treated for an extended period of time, waiting for transportation to a hospital or may stay at the clinic for up to 48 hours if they do not need hospital services but require monitoring and observation. The resources required to provide care during these encounters – including appropriate staffing available for call 24/7; capacity to call an extra provider in during normal clinic hours for emergency services; and space that can safely house a patient overnight – are challenges that every FESC must address. Encounters of 4–48 hours are considered extended-stay encounters.

Each FESC site recorded every extended-stay patient encounter via an On-line Clinical Outcome Log, which was developed by the Alaska Center for Rural Health (ACRH), Department of Nursing, University of Alaska Anchorage in concert with the FESC Consortium Steering Committee and the FESC project's Provider Workgroup.

Raw Outcome Log data were submitted via the Internet by the clinics on a Microsoft Access Outcome Log form. The data were downloaded by the Institute of Social and Economic Research (ISER), University of Alaska evaluation staff, into Statistical Package for Social Sciences (SPSS) for data coding, cleaning and analysis. Cleaned, analysed data were then transferred to Microsoft Excel to create the tables and figures for analysis. The extended-stay services data from the FESCs are for the period 15 September 2005 to 14 September 2010.

The chief complaints as reported by the patient at the time of admission to the clinic and the diagnosis as determined by the medical provider at the time of discharge were collected in the outcome log. Patients’ chief complaints and provider diagnoses were recorded in a fill-in-the-blank format; the researchers, in close consultation with the Provider Workgroup leader, recoded these open-ended answers into closed-ended categories. The answers were placed into a single, “best fit” category rather than into multiple categories.

Specific destination communities of the medevacs (patients transported to another facility) were recorded in the Outcome Log form. If the destination was not recorded, it was classified “unspecified.” Monitoring and observation encounters that ended as transfers were coded as such.

## Findings

### Number and duration

A total of 2,226 extended-stay encounters of all 5 facilities – ≥4 hours – were analysed, representing extended-stay encounters between 15 September 2005 and 14 September 2010, for Alicia Roberts Medical Center (ARMC), Cross Roads Medical Center (CRMC), IIMC, and lliuliuk Family Health Services (IFHS); and between 15 September 2006 and 14 September 2010, for HMC which entered the project later than the others. Thus, the data set for each clinic includes 4 or 5 years of extended-stay encounters, capturing important seasonal variations, such as the fishing season in Unalaska, Alaska, where IFHS is located; and tourist season in both Friday Harbor, Washington, served by IIMC, and Glennallen, Alaska, home to CRMC. With multiple years of data we can see the emergence of patterns and trends among the 5 clinics.

Clinics reported the following total number of extended-stay encounters of ≥4 hours for 5 years: ARMC, 706; CRMC, 384; IFHS, 830; IIMC, 74; and HMC, 232 (for 4 data years). Over the entire data collection period, the mean duration of an extended-stay encounter was 9.1 hours (see Table I).

**Table I:** Mean duration, Medicare and Medicaid eligible of extended-stay encounters by FESC site 2005–10

		Mean duration in hours						
						
FESC sites	Total extended-stay encounters	All encounters	Monitoring and observation	Transfers	Number of Medicare eligible	Percent Medicare eligible	Number of Medicaid eligible	Percent Medicaid eligible
ARMC Klawock, AK	706	8.5	9.2	7.2	250	35.4	101	14.3
CRMC Glennallen, AK	384	10.9	12.8	6.8	109	28.4	59	15.4
HMC (2006–10) Haines, AK	232	7.6	7.4	7.6	105	45.3	15	6.5
IFHS Unalaska, AK	830	9.6	8.7	11.1	101	12.2	23	2.8
IIMC Friday Harbor, WA	74	5.5	5.1	6.3	33	44.6	4	5.4
All sites	2,226	9.1	9.5	8.6	598	26.9	202	9.1

### After business hours

All of the clinics experienced many extended-stay encounters that were initiated or continued after normal business hours (see Table II). Distribution of encounter types in all clinics, however, did not vary appreciably between those occurring during regular operating hours and those occurring after hours, showing that patient classification and treatment decisions were not associated with the timing of the start extended-stay encounter. Over the 5 data years, 45.9% of the project's encounters commenced after hours, with 53.4% at CRMC, 47.3% at ARMC, 48.3% at HMC, 23.0% at IIMC and 39.4% at IFHS. Thus, all clinics experienced almost a quarter to over half of the FESC workload that started or extended to after the normal business hours.

**Table II:** FESCs normal business hours

FESC	Operating schedule
ARMC, Klawock, AK	Mon.–Fri. 8 a.m.–5 p.m.; Wed. 1 p.m.–5 p.m.; Sat. & Sun. – closed
CRMC, Glennallen, AK	Mon. 9 a.m.–5:30 p.m.; Tues., Wed., Fri. 10 a.m.–4:40 p.m.; Thurs. 1 p.m.–7:30 p.m. (starting Oct 1st, 2007 10 a.m.–7:30 p.m.); Sat. (starting Oct 1st, 2007 10 a.m.–2 p.m.); Sun. – closed
HMC, Haines, AK	Mon.–Fri. 8 a.m.–5 p.m.; Sat. & Sun. – closed
IFHS, Unalaska, AK	Mon.–Fri. 8:30 a.m.–6 p.m.; Sat. 8:30 a.m.–5 p.m.; Sun. – closed
IIMC, Friday Harbor, WA	M–F 8 a.m.– 5 p.m.; Sat. 10 a.m.–1 p.m.; Sun. – closed

### Chief complaints

**Fig. 1:**
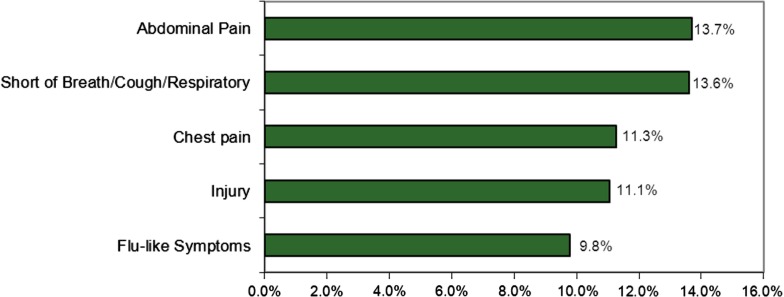
Percentage of FESC extended-stay encounters by 5 most-frequent chief complaints 2005–10.

The 5 most frequent chief complaints reported by patients for extended-stay encounters were 59.4% of the total over 5 years. Abdominal pain (13.7%, *n*=305) was the leading chief complaint. The other 4 most frequently reported complaints were: shortness of breath/cough/respiratory symptoms (13.6%, *n*=303); chest pain (11.3%, *n*=251); injury (11.1%, *n*=246); and flu-type symptoms, as described by patients, such as nausea, diarrhoea, vomiting, etc. (9.8%, *n*=218) (see Fig. 1). Other chief complaints reported less frequently included dizziness/syncope/confusion (6.3%, *n*=140); behavioral/mental health complaints (5.5%, *n*=123); and fever (3.7%, *n*=82).

### Discharge diagnoses

**Fig. 2:**
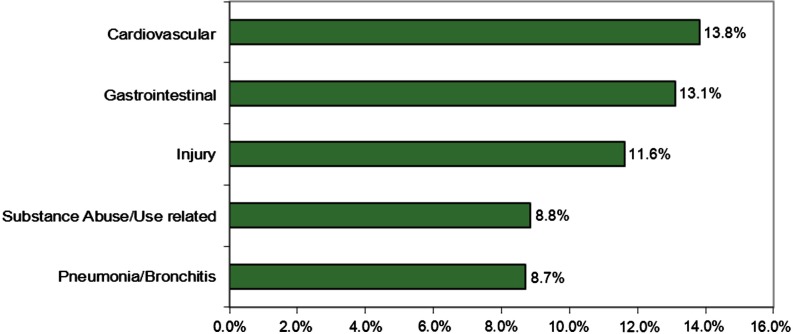
Percentage of FESC extended-stay encounters by 5 most-frequent discharge diagnoses 2005–10.

The 5 most common diagnoses at discharge for FESC patients as determined by the medical provider represent 56.2% of all diagnoses. Cardiovascular diagnoses were the most frequent, (13.8%, *n*=308), followed by gastrointestinal (13.1%, *n*=292) and injury (11.6%, *n*=259), substance abuse (8.8%, *n*=197) and pneumonia/bronchitis (8.7%, *n*=194) (see Fig. 2). Other less-frequent diagnoses at discharge included renal/urinary (6.3%, *n*=140), respiratory (6.2%, *n*=139) and neurologic injury/problem (3.5%, *n*=77).

**Fig. 3:**
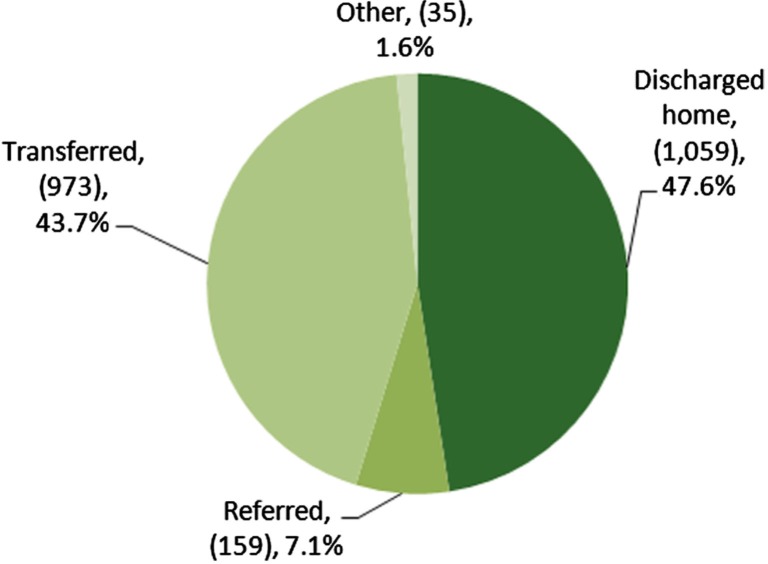
Discharge disposition of FESC extended-stay encounters 2005–10.

Over 5 years, 47.6% of extended-stay encounters (*n*=1,059) were discharged home. Another 43.7% of the encounters (*n*=973) were transferred. A little more than 7% (7.1%, *n*=159) were referred to another health facility for non-urgent follow up. The small “Other” category (1.6%, *n*=35) included a variety of dispositions, such as patients who refused to be transferred; patients who arranged their own transportation for transfer; patients referred to long-term care facilities, prisons and women's shelters; aborted medevac flights; deceased patients; and patients for whom there were no data (see Fig. 3).

### Transfer destinations

Transfer destinations depended upon the location of the clinic. IIMC in Friday Harbor, Washington transports to multiple Puget Sound destinations while IFHS and CRMC move the vast majority of their patients to Anchorage, ARMC (located in Southeast Alaska) transfers to multiple sites that include Sitka and Ketchikan as well as Anchorage, all in Alaska, and Seattle, Washington. HMC located in southeast Alaska transfers primarily to Juneau, Sitka and Anchorage, Alaska. Anchorage received 52.0% (504/970) of all transfers. Only 5.5% (*n*=68) of the transfers over 5 years used paid escorts to accompany patients during transport.

### Medicare and Medicaid eligibility

**Fig. 4:**
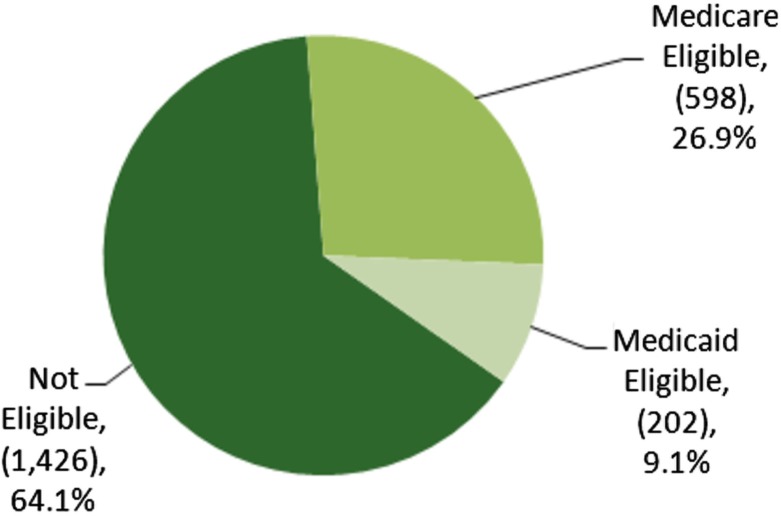
Percent of FESC extended-stay encounters eligible for Medicaid and Medicare 2005–10.

Over the 5 data years, 26.9% of total extended-stay encounters (598/2,226) were eligible for Medicare payment (see Fig. 4). This varied from clinic-to-clinic; IIMC had the largest portion of the encounters with Medicare as the primary payer at 44.6% (33/74) and IFHS, with its large working-age population, had only 12.2% (101/830) (see Table I).

In contrast, only 9.1% (202/2,226) of extended-stay encounters had Medicaid as the primary payer (see Fig. 4). CRMC had the highest percentage at 15.4% (59/384) and IFHS with the lowest percentage at 2.8% (23/830) (see Table I).

### Extended-stay encounters by type

Extended-stay encounters can be described by mainly 2 types: transfers and monitoring and observation. A transfer encounter occurs when a patient is transferred to another facility, an acute-care hospital, or critical-access hospital. Wait time can be as little as 1/4 hour or as long as 3 days because of adverse weather conditions or other circumstances which limit or prevent such direct transportation. Most other encounters involve monitoring and observation. Additionally, a small number of other encounters conclude when the patient expired; patient refused medevac; patient left clinic against medical advice; patient stabilized before transfer to long-term care facility, women's shelter, or incarceration; patient transport denied by the receiving hospital; or patient declined medevac and used his/her own transportation.

The overall monitoring and observation, and transfer mean durations are within 1 hour of each other −9.5 hours for monitoring and observation and 8.6 hours for transfer. A closer look, clinic-by-clinic reveals there are some slight differences between the overall, monitoring and observation, and transfer means. CRMC was characterized by the longest mean encounters for overall extended-stay encounters at 10.9 hours. The longest transfer mean −11.1 hours – was at IFHS (see Table I).

Monitoring and observations accounted for 60.9% (1,355/2,226) of the project's overall encounters. Four of the 5 clinics had a similar percentage of monitoring and observation encounters: ARMC 63.3% (447/706); CRMC 66.4% (255/384); IFHS 62.7% (520/830); and IIMC 64.9% (48/74). Only HMC had a significantly smaller percentage at 36.6% (85/232).

**Fig. 5:**
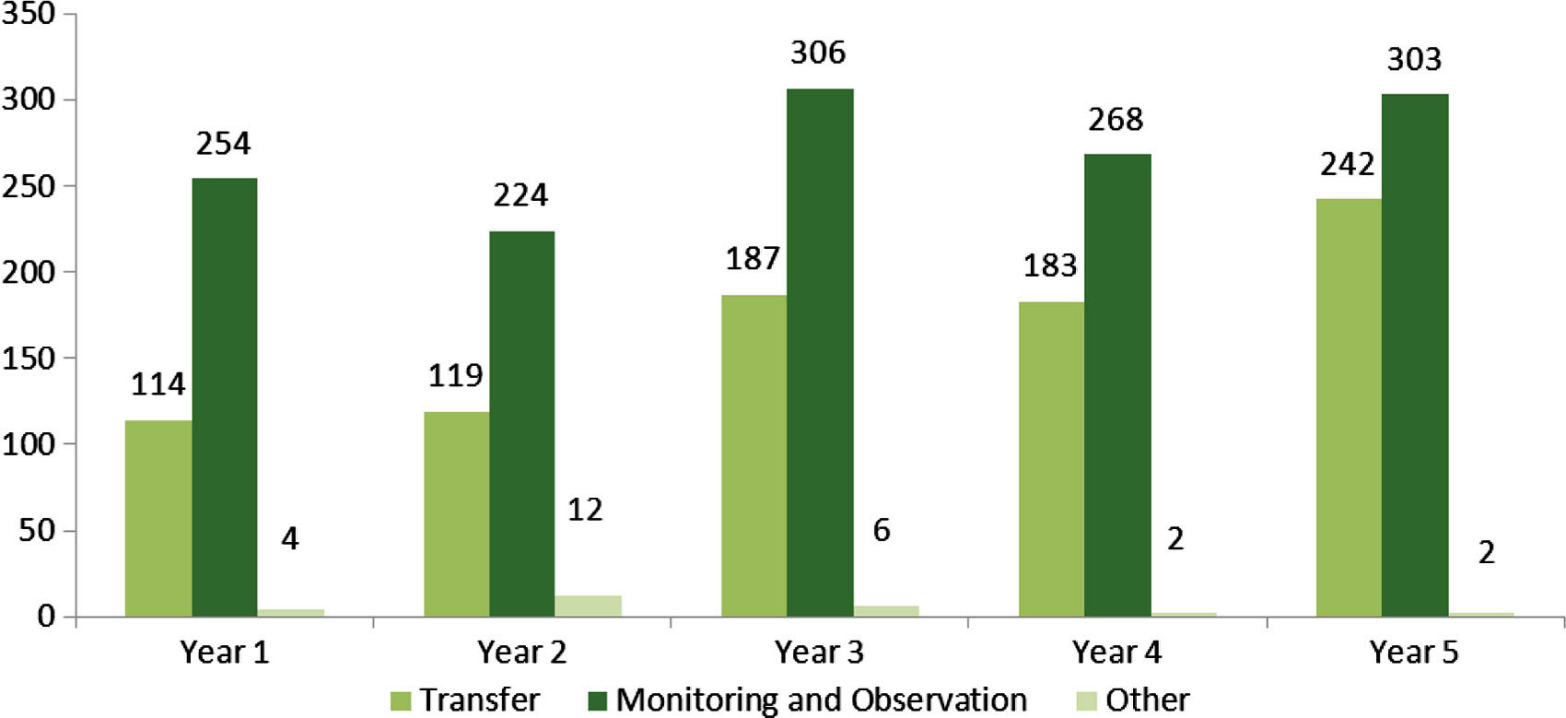
Number of FESC extended-stay encounters by type and data year.

The percentage of monitoring and observation encounters has decreased each year over the 5 years of the demonstration from 68.3% (254/372) in year 1 to 55.4% (303/547) in year 5, while the percentage of transfers has increased from 30.6% (114/372) in year 1 to 44.2% (242/547) in year 5. However, cumulatively over 5 years, 60.9% (*n*=1,355) of extended-stay encounters were designated monitoring and observation and 38.0% (*n*=845) of the 2,226 extended-stay encounters were designated transfers. A marginal percentage (1.1%, *n*=26) was designated “Other” (see Fig. 5). While transfer and monitoring and observation encounters have increased over 5 years, transfers have increased more rapidly than monitoring and observation. The trend is not significant for monitoring and observation encounters. However, this increase is a significant upward trend in the number of transfer encounters over the 5 years of the project with *P*=0.014.

## Limitations

The FESC project chose a documentation approach that yielded summary information for the demonstration of this model. The emphasis of the documentation was the effectiveness, appropriateness, viability and sustainability of the new facility type. Therefore, the findings for the number and duration of encounters, after-hours encounters, chief complaints, diagnoses, transfers, Medicare/Medicaid eligibility and extended-care types are descriptive. Because this is a new facility type, there is a lack of studies with which we could compare or contrast these data. Given the lack of studies and the differences between these new extended stay clinics and the other types of facilities for which performance metrics have been established, FESC project staff felt it was premature to establish performance measures or attempt to compare performance between FESC sites or between extended stay clinics and other facility types. Future research should clarify the benefits and limitations of this type of facility and identify contextual and other factors that affect facility performance. Such research could lead to useful performance metrics and greater clarity on the full range of data needed to assess extended stay clinic performance. However, the current data has limited utility for judging performance.

We do not have contextual data to determine if the trend of transfers will continue to climb, level out, or decline. The total number of extended-stay encounters increased over the 5 years of the project. We lack data on the total volume of encounters to determine the ratio of extended-stay to non-extended-stay encounters at these facilities. We need more information such as total encounters (extended stay and non-extended stay) by year for each facility and service area population to draw additional conclusions from these findings. There is also a need to study other characteristics of this new facility model. One would be to explore if there was a reduction of unfavourable outcomes relative to the provision of services through the FESC model.

## Conclusion

The importance of this project is the experience of these clinics as a foundation for the recognition of the FESC as a “New Provider Type” by Centers for Medicaid and Medicare (CMS). The term “Provider Type” is used by CMS to identify health care providers that receive payment through Medicaid and Medicare for services provided to beneficiaries. In addition to payment from Medicaid and Medicare, this recognition often leads to the acceptance of a “Provider Type” by private third party payers. This article provides a descriptive overview of the clinical services, disposition of patients, and potential Medicare and Medicaid eligible encounters for FESC. It shows that there is potential to provide a wider array of services in a patient's home community.

The distances and sparse population in remote and frontier areas make access to health care difficult. Many of these areas can support a clinic, a doctor's office, or a similar facility for primary care; but there is an on-going need for these facilities to provide emergent and extended-stay care as patients present themselves. The FESC facility model is one way for primary-care facilities to expand their scope of services. This type of facility model offers people in remote and frontier areas a facility that can offer consistent-quality primary, emergent and extended-stay care.

## References

[1] US Department of Health and Human Services, Health Resources Administration, and Office of Rural Health Policy (2006).

[2] National Center for Frontier Communities (2000). Extended stay primary care working with frontier communities to design facilities that work. http://www://frontierus.org/extended.htm.

[3] National Center for Frontier Communities (2006). UPDATE Frontier Extended Stay Clinic (FESC) summer. http://www.frontierus.org/policy.htm.

[4] Wakerman J (2004). Defining remote health. Aust J Rural Health.

[5] Salomone J (2006). Prehospital triage of trauma patients: a trauma surgeon's perspective. Prehosp Emerg Care.

[6] Stamp G, Miller D, Coleman H, Milera A, Taylor J (2006). They get a bit funny about going – transfer issues for rural and remote Australian Aboriginal people. Rural Remote Health.

